# Improving Dietary Guidance for Older Americans: Bridging the Research Gaps

**DOI:** 10.1093/geroni/igaf122.1870

**Published:** 2025-12-31

**Authors:** Kyla Shea, Sarah Booth, Sameera Talegawkar

## Abstract

The Scientific Report of the 2025 Dietary Guidelines Advisory Committee was recently released (in December 2025). This report is a vital resource in shaping the Dietary Guidelines for Americans, which are updated every five years. It highlighted older adults as a key population requiring updated dietary guidance to support their health and longevity. However, several critical knowledge gaps were identified regarding how to best address older adults’ unique nutritional needs. For example, frailty and cognitive impairment are increasingly common in older age, yet there remain significant gaps in our understanding of the specific role diet plays in either mitigating or exacerbating these conditions. Furthermore, studies that focus on racially and ethnically diverse populations are limited. These gaps must be addressed before dietary recommendations for older adults can be appropriately tailored. Meal timing has emerged as an important dietary factor to consider for healthy aging. Yet, the impact of meal timing on lifespan is uncertain. This is an important question to clarify to begin to develop reliable guidance about meal timing. Little is known about how diet quality varies among racial and ethnic groups across older adulthood, highlighting a gap in developing culturally appropriate dietary recommendations to improve diet quality in older age. This symposium aims to present and discuss recent research that addresses these gaps in our understanding of the dietary needs of older adults to help inform the development of evidence-based dietary guidelines that can support the health and well-being of older adults from diverse backgrounds.

